# Negligible contribution of M2634V substitution to ZIKV pathogenesis in AG6 mice revealed by a bacterial promoter activity reduced infectious clone

**DOI:** 10.1038/s41598-018-28890-0

**Published:** 2018-07-12

**Authors:** Fanfan Zhao, Yongfen Xu, Dimitri Lavillette, Jin Zhong, Gang Zou, Gang Long

**Affiliations:** Key Laboratory of Molecular Virology and Immunology, Institut Pasteur of Shanghai, Chinese Academy of Sciences, Shanghai, China

## Abstract

ZIKV has emerged as a significant human pathogene for the severe neurological complications, including Guillain-Barré(GBS) syndrome in adults and a variety of fetal abnormalities such as microcephaly. A stable and efficient infectious clone of Brazilian ZIKV isolate is required to study pathogenesis of epidemic ZIKV and virus evolution impact on it. Here we successfully constructed infectious cDNA clone on an early Brazilian isolate by eliminating the activity of predicted bacterial promoter in 1–3000 nt of ZIKV genome, leading to a stable infectious cDNA clone (pZL1). pZL1 derived virus could infect different cell lines and cause lethal effect to AG6 mice. We further investigated the role of a recent emerged substitution in NS5 (M2634V). We found that a reverse mutation (V2634M) caused negligible effect on the ZIKV viral genome replication and infectious progeny production in multiple cell culture systems. Additionally, this mutation did not alter the pathogenesis feature and virulence of ZIKV in AG6 mice. In summary, our results present another robust infectious ZIKV clone from Brazilian isolate and provide evidences to support that M2634V single mutation did not alter virus life cycle in cell culture and pathogenesis in AG6 mouse model.

## Introduction

Zika virus (ZIKV) belongs to the Flavivirus genus of the Flaviviridae family, which includes other globally relevant arthropod-transmitted human pathogens such as dengue virus (DENV), yellow fever virus (YFV), West Nile virus (WNV), Japanese encephalitis virus (JEV), and tick-borne encephalitits virus (TBEV)^1,2^. Within the mosquito-borne clade of flaviviruses, ZIKV is a member of the Spondweni group^3^. ZIKV is an enveloped positive-strand RNA virus. Its genome (approximately 10.8 kb in length) encodes a single polyprotein, which is cleaved co- and post-translationally by host and viral proteases into three structural proteins (capsid, pre-membrane, and envelope) and seven nonstructural (NS) proteins (NS1, NS2A, NS2B, NS3, N4A, NS4B and NS5). The structural proteins are the building blocks for producing infectious progeny virus, whereas the NS proteins are responsible for viral genome replication and in addition, to dampen host antiviral responses^4–6^.

ZIKV was first isolated in 1947 from a febrile sentinel rhesus monkey in the Zika forest in Uganda. In the same forest, ZIKV was subsequently isolated from *Aedes africanus* mosquitoes, but not from other animals, suggesting that primates are the primary vertebrate hosts for ZIKV^7^. In the decades following the discovery, ZIKV was only sporadically isolated from humans during outbreaks in Africa and Southeast Asia^8–12^. There, ZIKV infection has been associated with a self-limiting febrile and mostly asymptomatic illness. In 2007 however, ZIKV raised global attention because of pandemic spread, starting off with an explosive outbreak in Yap Island, Micronesia where approximately 75% of the population became infected within a 4-month period^3^. Between 2013 and 2014, a ZIKV outbreak in French Polynesia was the first to reveal an association between ZIKV infection and congenital syndrome, Guillain-Barre syndromé (GBS) and microcephaly^13,14^. In early 2015, ZIKV was detected in Brazil with more severe neurological complications. During this epidemic ZIKV infection has been linked to a drastic increase of severe fetal abnormalities that include spontaneous abortion, stillbirth, microcephaly and placental defect^15–22^. These data suggested that the severe neurological complications of ZIKV infections (including GBS and microcephaly) are associated with recently emerged ZIKV strains.

Because these severe ZIKV infection outcome and the magnitude of the outbreaks, researches are undergoing to understand the mechanisms responsible for the dramatic emergence of ZIKV. Very recently, experimental evidences were shown to support a view that genetic changes during ZIKV evolution caused the dramatic emergence and neuroinvasiveness of ZIKV. A982V substitution located in NS1 significantly improved ZIKV transmission efficiency from humans to mosquitoes through enhanced NS1 secretion^23^. S139N substitution located in prM substantially increased ZIKV infectivity in both human and mouse neural progenitor cells which led to more significant microcephaly in mouse fetus^24^. These two mutations (A982V and S139N) were emerged before 2014. Another more recent substitution M2634V located in NS5 likely occurred after ZIKV spread to Latin America^25^. This mutation is located in the interface between methyltransferase domain and RNA-dependent RNA polymerase domain, which leads to our hypothesis that substitution M2634V might alter ZIKV genome replication efficiency. Additionally, whether this substitution contribute to the recognized increased incidence of microcephaly in Latin America requires experimental evidence. Therefore, a reverse mutation V2634M on ZIKV Brazil isolate is necessary to clarify these points.

Virus infectious clone is a powerful tool for the understanding of virus evolution, transmission, pathogenesis and immune response. Compared to *de novo* generation of ZIKV isolates^26^, generation of reverse genetics systems for flavivirus was not often straightforward by using traditional clone methods because of common instability of flavivirus cDNA in *Escherichia coli* host^27^. Several cloning strategies have been used for developing ZIKV infectious clone, such as high fidelity low-copy number vector, introns insertion, multi-piece ligation system and bacterium-free approaches^28–33^. In this study, we developed a full-length cDNA clone of ZIKV (SPH2015, early Brazil strain) by eliminating the activity of predicted bacterial promoter in 1–3000nt of ZIKV genome (KU321639). We found this infectious clone (named pZL1) was stable in bacteria, and the cDNA clone-derived ZIKV virus could infect a panel of cell lines including Vero E6, C6/36, U-251MG and cause AG6 mice lethal effect. Furthermore, we took advantage of this infectious clone to analysis the role of V2634M reverse mutation in ZIKA infectivity on cell culture and mouse model. Results showed that V2634M substitution caused negligible effect to ZIKV life cycle in cell culture and pathogenesis in AG6 mice.

## Materials and Methods

### Cell lines, Virus and Antibodies

African Green kidney cell line Vero E6, human glioblastoma U-251 MG and human lung carcinoma cell line A549 cell lines were cultured in Dulbecco’s modified minimal essential medium (DMEM; Invitrogen) supplemented with 10% fetal calf serum and 2 mM L-glutamine, nonessential amino acids, 100 U penicillin per ml, 100 µg streptomycin per ml at 37 °C with 5% CO2. *Aedes albopictus* C6/36 cells were grown in minimum essential medium (MEM, Invitrogen) with 10% fetal calf serum and 2 mM L-glutamine, nonessential amino acids, 100U penicillin per ml, 100 µg streptomycin per ml at 28 °C with 5% CO2.

The ZL1 virus (Genbank number: KU321639.1) was rescued by a stabilized ZIKV infectious cDNA clone pZL1 (in this study). And ZIKV SZ-WIV01 strain (GenBank number: KU963796) was obtained from the Center for Emerging Infectious Diseases (Wuhan Institute of Virology, Chinese Academy of Sciences, Wuhan, China). The titers of ZIKV produced in different cell lines were measured by plaque-forming units (PFU) assay on Vero E6 cells.

Rabbit polyclonal antibody of ZIKV viral proteins (C, E, NS1, NS3, NS5) were generated in house. The following commercial antibodies have been used in this study: A mouse monoclonal antibody 4G2 cross-reactive with flavivirus E protein (Merck Millipore), mouse anti-STAT2(Santa-cruz), mouse anti-tubulin (Santa-cruz), mouse anti-actin (Santa-cruz), and goat anti-mouse IgG conjugated with Alexa Fluor 488 (Thermo Fisher scientific).

### Construction of infectious cDNA clone and related subgenomic replicon

Artificially synthesized ZIKV cDNA sequence 1(1–5972 bp from KU321639) including a T7 promoter and sequence 2 (5973–10379 bp from KU321639 plus 10380–10808 from KU527068) including a ribozyme sequence were designed to replace HCV sequence into low-copy plasmid pFK-Jc1E2Flag by restriction endonuclease (Sbf1/Afe1/Mlu1; New England Biolabs)^34^. The final full-length ZIKV infectious cDNA clone, named as pZL1, was validated by sequencing. Furthermore, a single amino acid mutation (V2634M) was inserted by a standard overlap PCR. Briefly, two DNA fragments harboring the substitution were fused by overlap PCR. PCR amplified sequence was then subclone in pZL1 between the two restriction endonuclease site [RsrII(7473–7479 bp)/MluI] to generate the pZL1(V2634M).

For construction of ZIKV subgenomic replicon (SG), vast majority of structural proteins (39aa-764aa) were deleted and replaced by a Renilla luciferase gene and FMDV 2A gene (R2A)^29,35^. This replicon (pSGR) was also validated by DNA sequencing before applied for further experiments.

### RNA transcription and transfection

The full-length infectious clone pZL1, single amino acid mutated infectious clone pZL1 (M) and subgenomic replicon pSGR were amplified in competent *E. coli* Top10 and purified by Maxiprep (QIAGEN). All plasmids (10 µg) are linearized with restriction enzyme MluI before *in vitro* transcription. The linearized plasmids were extracted with phenol-chloroform and chloroform, precipitated with ethanol, and resuspended in 10 µl of RNase-free water. The mMESSAGE mMACHINE kit (Ambion) was used to *in vitro* transcribe RNA in a 20 µl reaction with an additional 1 µl of 30 mM GTP solution. The reaction mixture was incubated at 37 °C for 2 hr, followed by the addition of DNase I to remove the DNA template. The RNA was precipitated with lithium chloride, washed with 70% ethanol, re-suspended in RNase-free water, quantitated and stored at −80 °C in aliquots. For transfection, approximately 5 µg of RNA was electroporated to 5 × 10^6^ Vero E6 cells in 0.4 ml of cytomix buffer (2 mM ATP; 5 mM glutathione; 120 mM KCl; 0.15 mM CaCl_2_; 10 mM K_2_HPO_4_/KH_2_PO_4_; 25 mM Hepes; 2 mM EGTA; 5 mM MgCl_2_, pH7.6) in 4 mm cuvettes with the GenePulser apparatus (Bio-Rad) at settings of 0.45 kV and 25 µF, pulsing three times, with 3 s intervals^29^. After a 10 min recovery at room temperature, the transfected cells were mixed with culture medium (3% FBS) and incubated in a 10 cm plate (5% CO_2_ at 37 °C). The virus supernatant was harvested twice at day 5 and day 8 post electroporation. This viral supernatant was clarified by centrifugation at 500 g for 10 min and then stored in aliquots at −80 °C.

### Plaque Assay

Viral samples were 10-fold serially diluted in DMEM. For each dilution, 200 µl sample was added to a 24-well plate containing Vero E6 cells at about 90% confluence. Infected cells were incubated for 1 hr and swirled every 15 min to ensure complete coverage of the monolayer for even infection. After the incubation, 1 ml of carboxymethyl cellulose overlay containing 1.5% FBS and 1% penicillin/streptomycin was added to each well, and the plate was incubated at 37 °C for 4 days. Following the incubation, methyl cellulose overlay was removed; the plate was washed once with PBS gently, fixed with 4% formaldehyde, and incubated at room temperature for 20 min. After removing the fixative, the plate was stained with 0.05% crystal violet for 30 min. Visible plaques were counted, and viral titers (PFU/ml) were calculated.

### Western blot analysis

Samples for Western blotting were denatured in Laemmli buffer (125 mM Tris/HCl, 2% (w/v) SDS, 5% (v/v) 2-mercaptoethanol, 10% (v/v) glycerol, 0.001% (w/v) bromophenol blue, pH 6.8) and separated by SDS 12% polyacrylamide gel electrophoresis. Proteins were transferred onto a polyvinylidene difluoride (PVDF) membrane. The membrane was blocked for 1 hour in PBS supplemented with 0.5% Tween (PBS-T) and 5% dried milk (PBS-M) at room temperature, and then incubation for 2 hrs with primary antibody diluted in PBS-M. Membrane was washed 3 times with PBS-T and incubated for one hour with horseradish-peroxidase-conjugated secondary antibody diluted 1:10,000 in PBS-T with 1% dried milk. Bound antibodies were detected after 3 times washing with the ECL reagent (PerkinElmer). The signal was captured by Tanon 5500.

### Indirect Immunofluorescence Assays

For detection of viral protein expression in ZIKV RNA-transfected Vero E6 cells or ZIKA virus infected cells, cells grown on glass coverslips were fixed in formaldehyde (2% wt/vol in PBS) after prior wash with PBS and incubated in blocking reagents (3% BSA and 0.05% tween-20 in PBS). After overnight incubation at 4 °C with antibody against E or NS5 and 1 hour incubation with at room temperature with AlexFluor-488-conjugated secondary antibody to mouse ((Thermo Fisher scientific)), cell nuclei were stained with DAPI. Images were taken and analyzed with Olympus IX53.

### Luciferase Assay

To investigate the replication of ZIKA virus RNA in cells, Vero E6 cells transfected with SGR RNA (5 µg) were seeded in a 24-well plate. At various time points, the cells were washed once with PBS and lysed using cell lysis buffer (Promega). The cells were scraped from plates and stored at −80 °C. Once samples for all time points had been collected, luciferase signals were measured in a luminescence microplate reader (Thermo scientific Varioskan Flash) according to the manufacturer’s protocol.

### Replication Curves

Subconfluent Vero E6, C6/36 and U-251 MG cells in 24-well plates were inoculated with either ZL1(V) or ZL1(V2634M) virus at an MOI of 0.01 in triplicate wells. Virus stocks were diluted in DMEM, 200ul virus mixtures was added to each well. After 4hrs attachment (5% CO_2_ at 37 °C for Vero E6 cells and U-251 MG, at 28°Cfor C6/36 cells), the inocula were removed. The cell monolayers were washed three times with 1 × PBS. Afterward, 1 ml DMEM medium containing 3% FBS and 1% penicillin/streptomycin was added to each well. The plates were incubated for up to 5 days. The medium was collected daily and subjected to plaque assay as described above.

### Real-time PCR analysis

For quantification of ZIKA RNA in cells or tissues, relative-quantitative real time PCR was measured. First, RNA from cells, tissue samples, blood was isolated using TRIzol (Invitrogen) according to the manufacturer’s protocol. ZIKV RNA levels were measured by a one-step quantitative real time reverse transcription polymerase chain reaction assay (qRT-PCR) using One-Step RT-PCR Master Mix (Toyobo). Primers were as follows: ZIKA Forward: 5′-ATTGTTGGTGCAACACGACG-3′ (ZIKV KU321639 genome sequence nucleotides 670–689); Reverse: 5′-CCTAGTGGAATGGGAGGGGA-3′ (ZIKV KU321639 genome sequence nucleotides 763–782). GAPDH Forward: 5′-GAAGGTGAAGGTCGGAGTC-3′; Reverse: 5′-GAAGATGGTGATGGGATTTC-3′; C6/36 -Actin Forward: 5′-TCC-GTCTGGACTTGGCCGGT-3′; Reverse: 5′-CGGCGGTGGCCATTTCCTGT-3′. IFNα Forward: 5′-CTTGTGCCTGGGAGGTTGTC-3′; Reverse: 5′-TAGCAGGGGTGAGAGTCTTTG-3′; IFNβ Forward: 5′-GACGCCGCATTGACC-ATCTA-3′; Reverse: 5′-TTGGCCTTCAGGTAATGCAGAA-3′;

### Mice study

AG6 mice were used to examine the virulence of wild type virus ZL1 and mutated virus ZL1(V2634M). 10-week-old AG6 mice were infected with 100 or 1000 PFU of virus via the intraperitoneal route. PBS was used to dilute the virus stocks to the desired concentration. Mock-infected mice were given PBS via the same route. Mice were weighed and monitored daily. Mice were bled via the caudal vein after being anesthetized every other day. After weight the blood, 1 ml Trizol was added into Blood collecting tubes and immediately stored at −80 °C for storage. We also collected the different tissues of mice at 6 days post challenge for monitoring ZIKV infection tissue tropism.

### Accordance

All methods were carried out in accordance with relevant guidelines and regulations. Experimental protocols related to animal study and ZIKV infection were approved respectively by the Institutional Animal Care Committee and Bio-Safety Committee of Institut Pasteur of Shanghai.

## Results

### Construction of full-length infectious cDNA clone of ZIKV(SPH2015)

**Figure 1:**
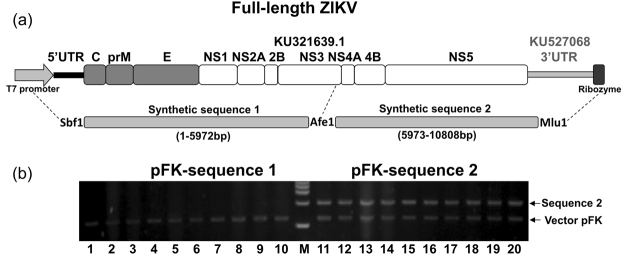
Schematic diagram of full-length ZIKV infectious cDNA clone (**a**) Artifical synthetic DNA sequences 1&2 that contain ZIKV cDNA and *in vitro* transcription elements (T7 promoter and Ribozyme). Full-length ZIKA cDNA was designed to construct into pFK vector by using indicated restriction enzymes (Sbf1/Afe1/Mlu1). (**b**) Artificial synthetic cDNA sequences 1&2 were inserted into low-copy plasmid pFK, respectively. After transformation, ten colonies (numbers as indicated) were picked for each and then purified plasmids were digested by restriction enzyme (Sbf1/Afe1 or Afe1/Mlu1), respectively.

Here, we chose an early Brazil autochthonous transmission strain to construct infectious clone. This strain (Genbank: KU321639.1) was isolated from a patient who received a blood transfusion from an asymptomatic donor at the time of donation in São Paulo state, Brazil, in 2015^36^. Due to incomplete sequence of its 3′UTR, we replaced its 3′UTR with another strain (KU527068.1) which was isolated from the brain of a fetus with microcephaly^37^. The ZIKV sequence was then synthesized artificially in two halves. As infectious clone backbone vector, we used a low-copy vector pFK which works efficiently for HCV infectious clone (Fig. 1a). The second half part (sequence 2) of synthetic ZIKV cDNA was easy to clone into pFK backbone, but we failed consistently to clone first half part (sequence 1) (Fig. 1b). We hypothesized that toxicity caused by putative bacterial promoters in ZIKV cDNA may be accountable for this^38^. Therefore, the first half part of ZIKA cDNA was subjected to analyzed for the prediction of putative bacterial promoters by using the Neural Network promoter program (http://www.fruitfly.org/seq_tools/promoter.html). As expected, 8 putative bacterial promoters with scores higher than 0.9 were found in ZIKA genome sequences 1–3000nt (cDNA) (Table 1).

**Table 1:** Prediction and silent mutation of bacterial promoter sequences within nt 1 to 3000 of the ZIKA (KU321639.1) genome.

Virus and *E. coli* promoter		Virus sequence (5′-3′)		Viral protein	Score
1 WT	798	GCTTTTGGGAAGCTCAACGAGCCAAAAAGTCATATACTTGGTCATGATAC	847	prM	0.98
Mutant		GCTTTTGGGTAGCTCAACGAGCCAAAAAGTCATATACTTGGTCATCATAC			ND
2 WT	1325	TCGTTAATGACACAGGACATGAAACTGATGAGAATAGAGCGAAGGTTGA	1373	E	0.97
Mutant		TCGTTAACGATACAGGACATGAAACTGATGAGAACAGAGCGAAGGTTGA			ND
3 WT	1729	GGCCACTTGAAATGTCGCCTGAAAATGGATAAACTTAGATTGAAGGGCG	1777	E	0.96
Mutant		GGCCACTTAAAGTGTCGCCTGAAAATGGACAAACTTAGATTGAAGGGCG			ND
4 WT	2098	GCATTTGAAGCCACTGTGAGAGGTGCCAAGAGAATGGCAGTCTTGGGAG	2146	E	0.94
Mutant		GCATTCGAAGCCACTGTGAGAGGTGCCAAGAGGATGGCAGTCTTGGGAG			ND
5 WT	2234	CATTGTTTGGAGGAATGTCCTGGTTCTCACAAATTCTCATTGGAACGTTG	2283	E	0.99
Mutant		CATTGTTCGGAGGAATGTCCTGGTTCTCACAAATTCTCATTGGAACGTTG			ND
6 WT	2565	AAGAATGGAAAACATCATGTGGAGATCAGTAGAAGGGGAGCTCAACGCA	2613	NS1	0.94
Mutant		AAGAATGGAGAACATCATGTGGAGATCAGTAGAGGGGGAGCTCAACGCA			ND
7 WT	2631	AGTTCAACTGACGGTCGTTGTGGGATCTGTGAAAAACCCCATGTGGAGAG	2680	NS1	0.90
Mutant		AGTTCAACTAACGGTCGTTGTGGGATCTGTCAAAAACCCCATGTGGAGAG			ND
8 WT	2838	CTTTCTTGTGGAGGATCATGGGTTCGGGGTATTTCACACTAGTGTCTGGC	2887	NS1	0.96
Mutant		CTTTCTCGTGGAGGATCATGGGTTCGGGGTGTTTCACACTAGTGTCTGGC			ND

The underlined nucleotides correspond to the mutated ones.

ND, not detectable.

**Figure 2:**
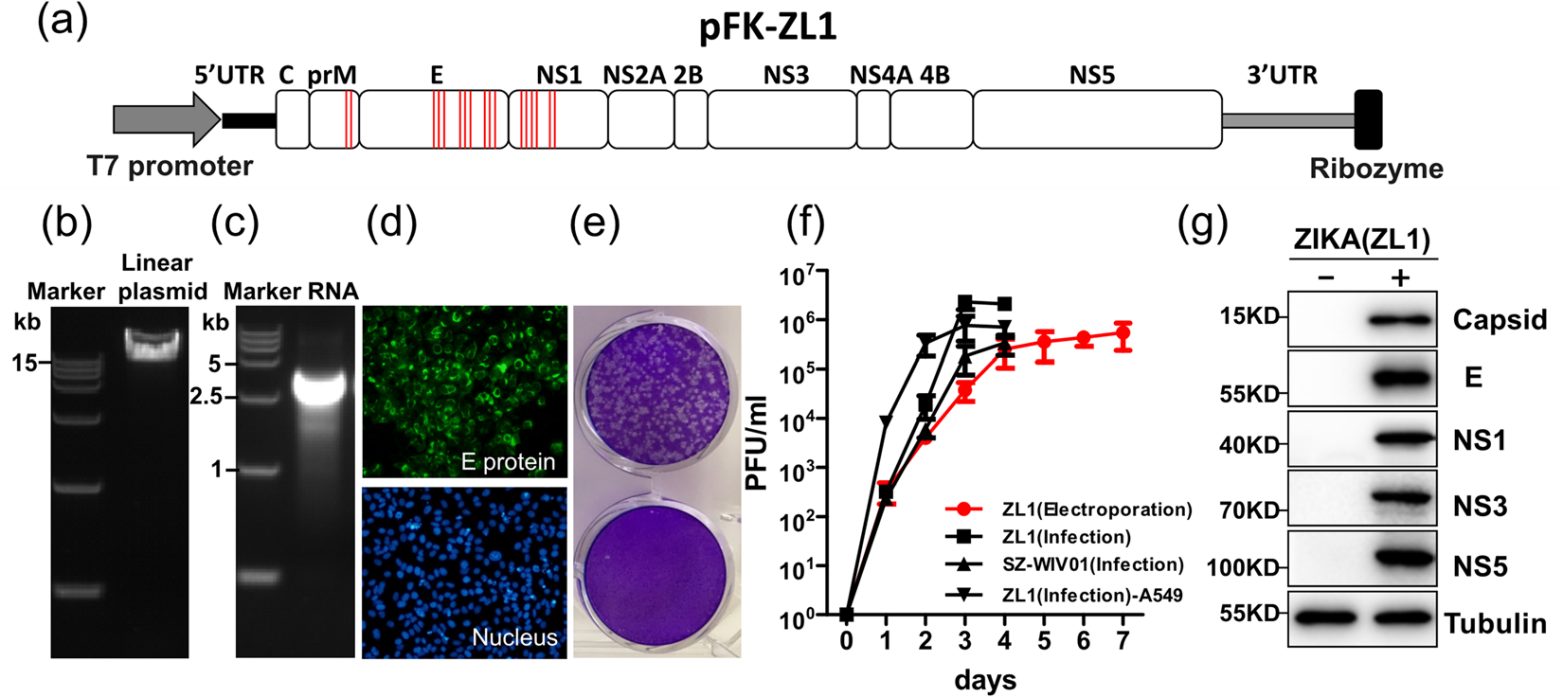
Construction of ZIKV infectious clone and virus rescue (**a**) Schematic diagram of full-length ZIKV infectious cDNA clone. 17 nucleotides of ZIKV sequence were mutated without altering the amino acid sequence to eliminate the activity of predicted bacterial promoters. Red bars in the box represent engineered mutation sites of ZIKV genome (Genbank: KU321639.1). (**b**) Analysis of linearized ZIKV infectious clone pZL1 on a 1% agarose gel. (**c**) Analysis of *in vitro* transcript from pZL1 on a 1% agarose gel. (**d**) Indirect immunofluorescence analysis of viral protein expression in Vero E6 cells after electroporation with full-length ZL1 RNA. Vero E6 cells were electroporated with 5ug of full-length ZL1 RNA. Cells were fixed and stained the viral E protein by using flavivirus antibody (4G2) at 5 days post electroporation. Green and blue represent E protein and nucleus (stained with DAPI), respectively. (**e**) Cytopathic effect (CPE) on Vero E6 cells at day 4 post infection. Fixed cells were stained by 0.05% crystal violet solution. (**f**) Growth kinetics of rescued ZL1 (electroporation), ZL1 (re-infection, MOI = 0.01), isolated strain SZ-WIV01 (infection MOI = 0.01) viruses in Vero E6 cells, or ZL1 (re-infection, MOI = 0.01) virus in IFN competent cell A549. Data represent the means of three independent assays; Error bars represent standard deviations from the means. (**g**) Western blotting analysis of expression of rescued ZL1 proteins. Vero E6 cells were infected with ZL1 virus (MOI = 0.01). Viral proteins were detected by using home-made polyclonal antibodies at 3 days post infection.

To reduce the bacteria toxicity likely caused by putative bacterial promoters in ZIKV genome, silent mutations were introduced during DNA synthesis to eliminate the activity of bacterial promoters (Fig. 2a, Table 1). The effects of all silent mutations were also evaluated with same website above mentioned to ensure the removal of bacterial promoter activities (Table 1). Following these modifications, the full-length ZIKV cDNA clone was constructed successfully and named pZL1 (Fig. 2a/b).

Based on the consensus view of instability and deletion of flavivirus infectious cDNA sequences in bacterial host, we next verified the stability of pZL1 in bacteria host by sequencing the whole cDNA after 5 continuous plasmid transformation (data not shown). These data showed that pZL1 is a stable ZIKC cDNA clone.

### Rescue of ZIKV from the full-length infectious cDNA clone pZL1

To test whether pZL1 could be used to rescue infectious ZIKV, *in vitro* transcripts from pZL1 was electroporated into Vero E6 cells to examine the infectious virion production (Fig. 2c). As shown in Fig. 2d, the transfected Vero E6 cells could express viral E protein efficiently at day 5 post electroporation. After harvesting the virus supernatant of transfected cells, the titer of rescued virus was detected by plaque assay. We could clearly observe cytopathic effects (CPE) staining by 0.05% crystal violet solution at day 4 post infection (Fig. 2e). Comparable to wild type ZIKV(SZ-WIV01) virus, infectious titer of rescue virus could reach a peak value of 2.7 × 10^6^ plaque-forming units(PFU) on day 4 post infection in VeroE6 cells (Fig. 2f). Several viral proteins (Capsid, E, NS1, NS3, NS5) could be detected by western blotting at day 3 post infection (Fig. 2g). Sequencing analysis of viral genome of ZL1 virus confirmed that the specific silent mutations of bacterial promoters still remained in ZL1 virus and no other mutation was detected (data not shown). Collectively, above results demonstrated that infectious ZIKV could be rescued by pZL1 cDNA clone.

### Virulence of ZL1 virus in AG6

**Figure 3:**
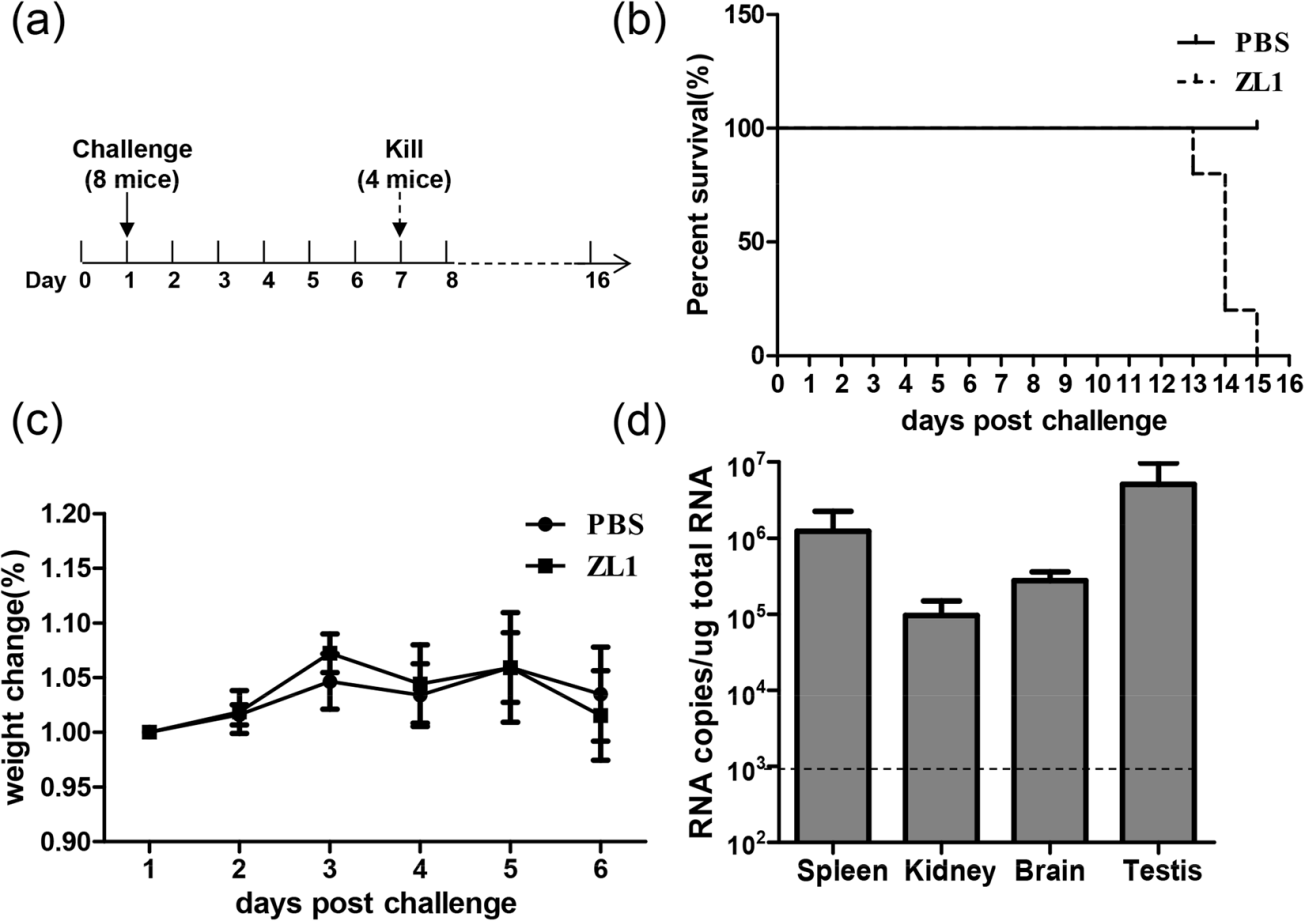
Virulence of Rescued ZL1 virus in the AG6 mice. (**a**) Schematic diagram of process of challenge in AG6 mice. 10-week-old AG6 mice were inoculated by intraperitoneal injection with 100PFU (n = 8), the infected mice were monitored for weight loss, and then, 4 mice were killed and several organs were collected at 6 days post infection for RNA analysis. (**b**) Survival rate of infected mice after inoculation with 100 PFU ZL1 virus. (**c**) Weights were obtained over 6 days and expressed as a percentage of starting weight. (**d**) RNA level in different organs of infected mice (n = 4). Several organs were collected at 6 days post infection, after RNA extraction from these organs, RNA copies were detected by q-RT-PCR. Data represent the means of 4 mice; error bars represent standard deviations from the means.

Having shown that ZL1 is infectious *in vitro*, we further tested its virulence *in vivo*. AG6 mice were inoculated by intraperitoneal injection with 100PFU (n = 8) (Fig. 3a). As shown in Fig. 3b, the injection of ZL1 virus could lead to death after 12 days, similarly to other previously reported ZIKV virus strains^32,33,39^. At day 6 post infection, half of the infected mice (n = 4) were killed for collection of organs, without observing significantly weight loss (Fig. 3c). ZIKV RNA was detected in spleen, kidney, brain and testis (Fig. 3d). These data confirmed that the tissue tropism of ZL1 virus is similar to the tropism of other previously described ZIKV strains.

### M2634V mutation in NS5 makes negligible contribution to ZIKV pathogenesis

**Figure 4:**
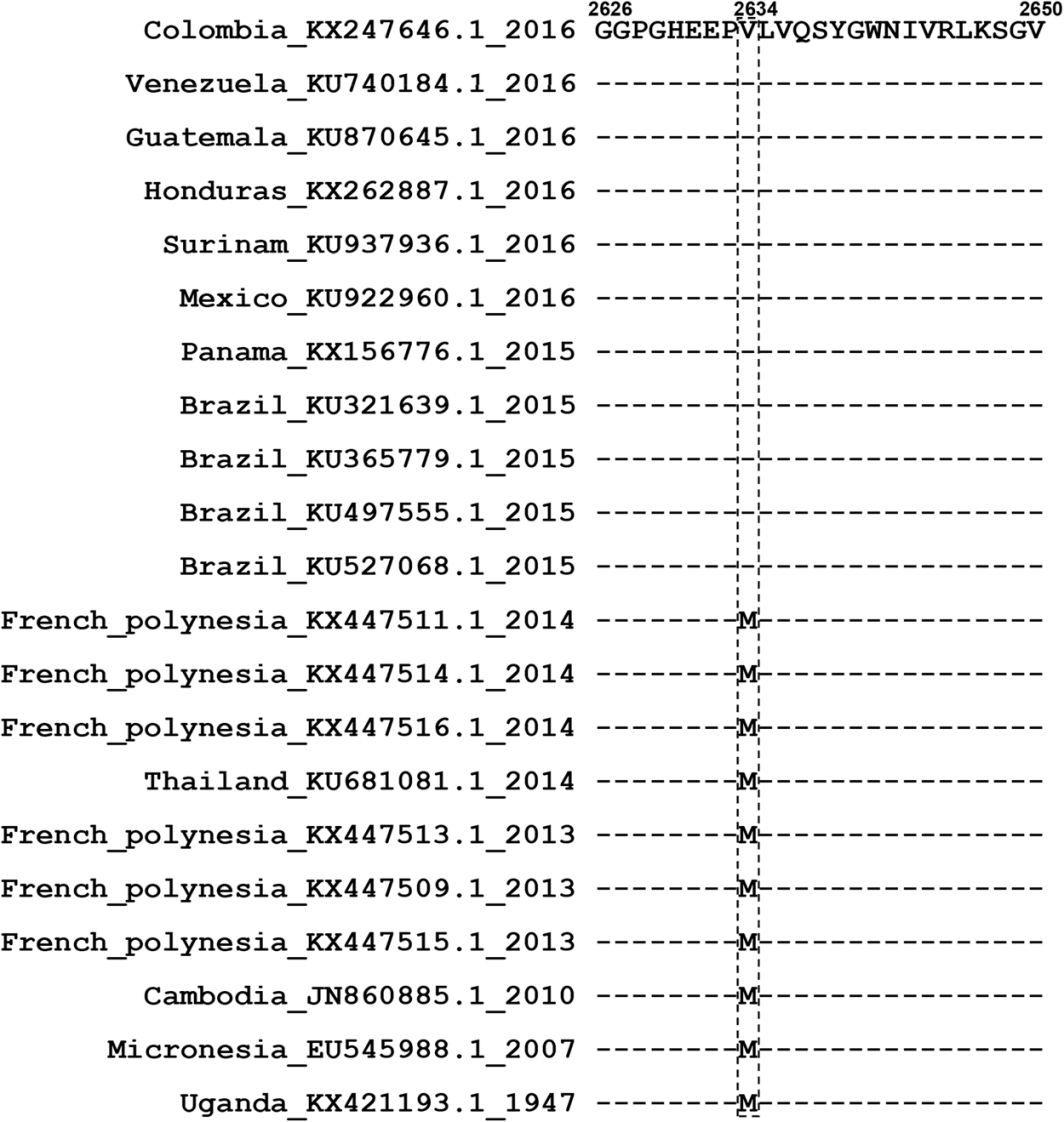
Sequence alignment of multiple ZIKA strains classified by time.

**Figure 5:**
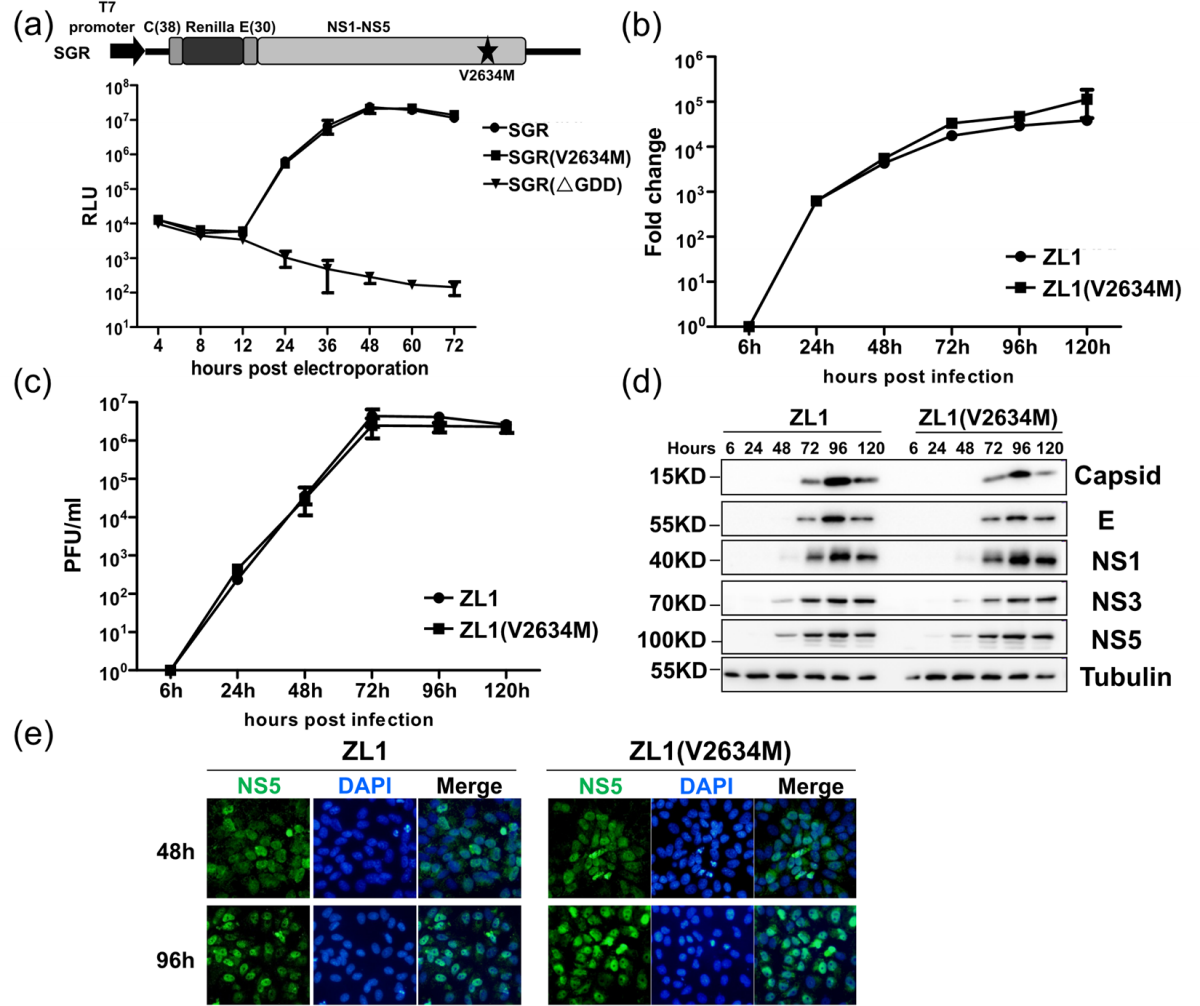
Comparison of the replication and infectivity of ZL1(V2634M) with wild type ZL1 in Vero E6 cells. (**a**) Replication analysis of wild type ZL1 with mutated ZL1(V2634M). The ZIKV subgenomic replicon with a *Renilla* luciferase (SGR, top) was engineered with mutation (V2634M). Equal amounts of ZL1 and ZL1(V2634M) replicon RNA (5ug) were electroporated into Vero E6 cells. Luciferase signals were measured at the indicated time points. A defective replicon containing an inactive NS5 polymerase with GDD-to-AAA mutation was included as a negative control. The averages of three replicates are presented. Error bars represent standard deviations from the means. (**b**/**c**) Comparison of the replication and virus production by wild type ZL1 and ZL1 (V2634M). Vero E6 cells were infected with ZL1 or ZL1(V2634M) at MOI = 0.01, the cell pellets and supernatants were collected at 6,24,48,72,96,120 hours post infection. Intracellular ZIKV RNA copies and titer of cell culture supernatant were detected by q-RT-PCR and plaque assay, respectively. Data represent the means of three independent assays; Error bars represent standard deviations from the means. (**d**) Western blot analysis of viral proteins in infected cells. The cell pellets were collected at different time points following infection (MOI = 0.01), and the levels of ZIKV protein (Capsid, E, NS1, NS3, NS5), STAT2 and Tubulin were analysed by western blot. (**e**) Nuclear localization of viral NS5 protein in Vero E6 cells. ZL1 and ZL1(V2634M) virus infected cells were fixed and stained with homemade Rabbit anti-NS5 antibody at 48 h or 96 h post electroporation. Green and blue represent NS5 and nucleus (stained with DAPI), respectively.

Previous phylogenetic analysis revealed that a specific amino acid mutation (M2634V), which was located in NS5 methyltransferase region near the interface between methyltransferase domain and RdRp domain, emerged after 2015 when ZIKA outbreaks in Brazil (Fig. 4)^25^. We hypothesized that this fixed mutation might play a significant role for ZIKV in increased human pathogenicity in 2015. Using pZL1 infectious cDNA clone, we further constructed a reverse mutation (V2634M) resulted in mutated ZIKV, ZL1(V2634M). To examine the impact of this substitution on translation and RNA synthesis, a ZL1-related subgenomic replicon with Renilla luciferase reporter gene (named as SGR) was constructed^29,35^. Data showed that replication kinetics of wild type SGR was similar to those of SGR(V2634M), indicating that this mutation did not affect ZIKV genome replication (Fig. 5a). Second, to examine whether this single amino acid mutation affect the virus life cycle in cell culture, we next compared the intracellular viral RNA level and infectious virions protduction of ZL1 and ZL1(V2634M) at different time points after infection of Vero E6 cells (MOI = 0.01). Results demonstrated that there were no significant differences between the wild type ZL1 and ZL1(V2634M) in virus replication and production (Fig. 5b and c). Additionally, western blot analysis showed that there was no significantly difference in viral protein expression between these two viruses (Fig. 5d). Moreover, confocal analysis showed that ZIKV V2634M mutation in NS5 protein caused no difference in subcellular localization pattern of NS5 (Fig. 5e)^40^.

**Figure 6:**
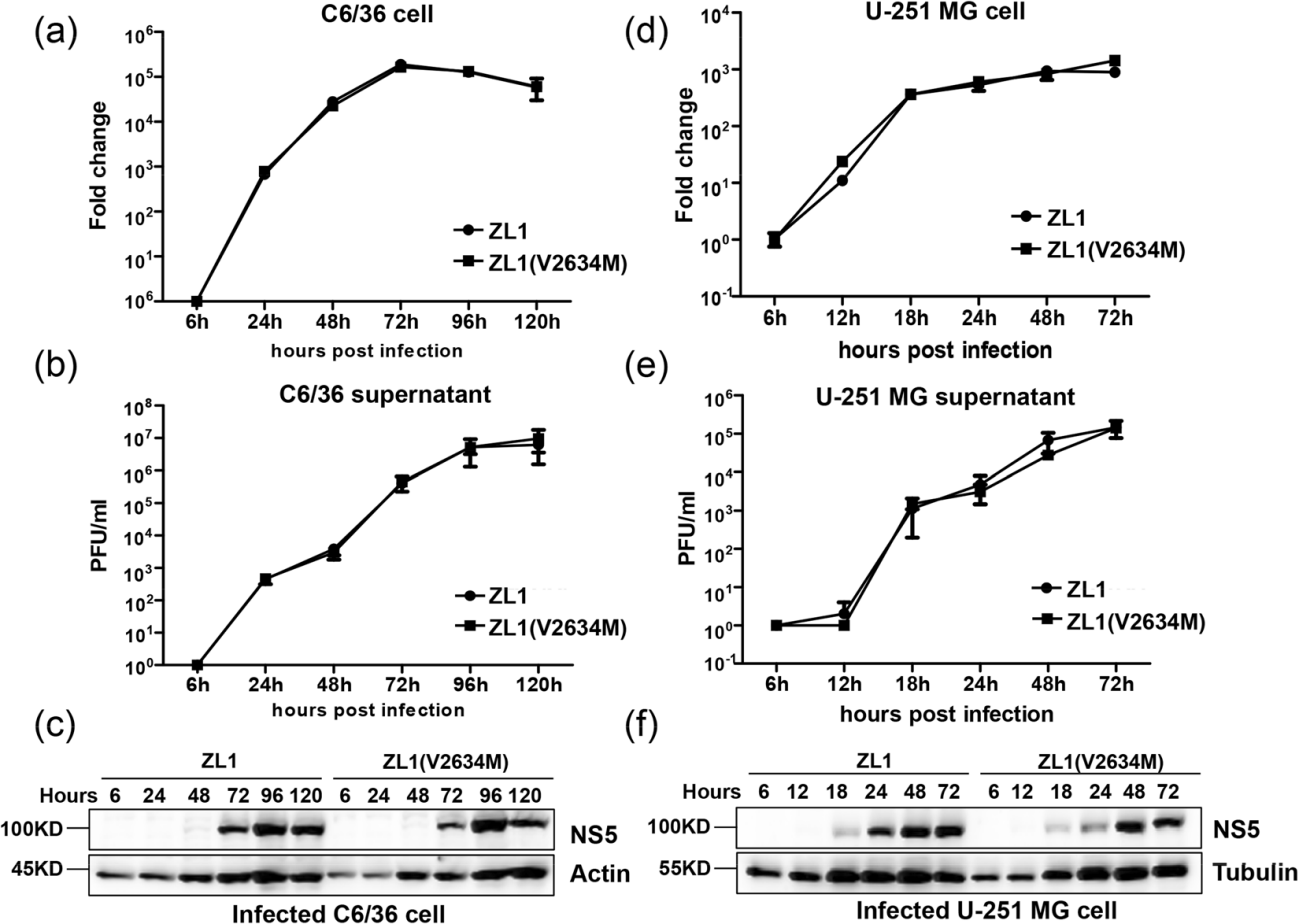
Comparing the replication and infectivity of ZL1(V2634M) with wild type ZL1 in C6/36 cells and U-251 MG cells. (**a**/**b**) Comparison of the replication and virus production of wild type ZL1 with mutated ZL1(V2634M) in C6/36 cells. C6/36 cells were infected with ZL1or ZL1(V2634M) at MOI = 0.01, the cell pellet and supernatant were collected at 6, 24, 48, 72, 96, 120 hours post infection. Intracellular ZIKV RNA copies and virus titer in cell culture supernatant were detected by q-RT-PCR and plaque assay, respectively. Data represent the means of three independent assays; Error bars represent standard deviations from the means. (**c**) Western blot analysis of viral protein in infected C6/36 cells. Cell pellets harvested at hours 6, 24, 48, 72, 96, 120 post infection were detected by using home-made polyclonal antibody against NS5. (**d**/**e**) Comparison of the replication and virus production of ZL1 and ZL1(V2634M) in U-251 MG cells. U-251 MG cells were infected with ZL1 or ZL1(V2634M) at MOI = 0.01, the cell pellet and supernatant were collected at 6, 12, 18, 24, 48, 72 hours post infection. Intracellular ZIKV RNA copies and virus titer in cell culture supernatants were detected by q-RT-PCR and plaque assay, respectively. Data represent the means of three independent assays; Error bars represent standard deviations from the means. (**f**) Western blot analysis of viral protein in infected U-251 MG cells. Cell pellets harvested at hours 6, 12, 18, 24, 48, 72 post infection were detected by using home-made polyclonal antibody against NS5.

Since ZIKV is transmitted by mosquito and caused drastic neurological complications in human, we hypothesized that M2634V substitution might contribute to ZIKV infection in mosquito cells or in neuron cell^39,41^. We then tested the infection of C6/36 cells (Ae. albopictus) and U-251 MG cells (human glioblastoma cell line) by ZL1and ZL1(V2634M) viruses. As shown in Fig. 6, both viruses had similar viral replication and virus production kinetics in both cell lines (Fig. 6). Interestingly, U-251 MG cells could support more rapid and efficient viral replication and expression of viral proteins than Vero E6 and C6/36 cells (Fig. 6d/e/f), suggesting a preferred fitness for human neuron cells^42,43^. Altogether, our data demonstrated that this reverse mutation V2634M hardly changed ZIKV life cycle in cell culture.

**Figure 7:**
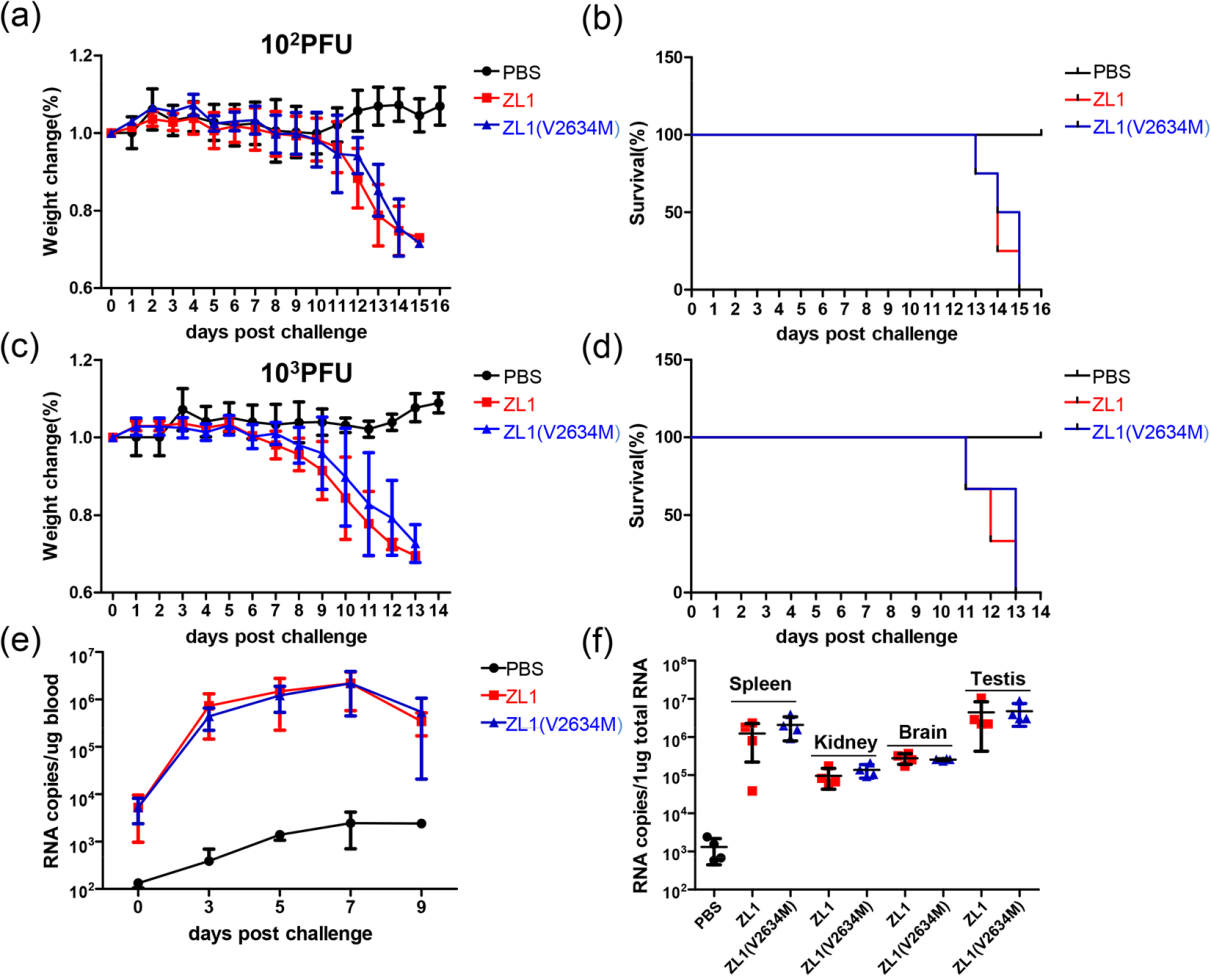
Comparison of virulence in AG6 mice between wild type ZL1 and ZL1(V2634M). (**a**/**b**/**c**/**d**) Challenge of AG6 mice with ZL1 and ZL1(V2634M). Ten-week-old AG6 mice were infected with 10^2^ PFU (**a**/**b**) or 10^3^PFU (**c**/**d**) per individual via the intraperitoneal route. Mock and infected mice (n = 4 per group) were monitored for weight loss. Weights were obtained over 6 days and expressed as a percentage of starting weight (**a**/**c**). Lethality was monitored for 15 days (**b**/**d**). (**e**/**f**) Analysis of ZIKV RNA levels in mice blood and tissues. Challenged mice (100 PFU, **a**/**b**) were bled via the caudal vein every other day and partial mice were killed for organs collection at day6 post challenged. RNA copies were detected by q-RT-PCR. Data represent the mean of 4 mice; error bars represent standard deviations from the means.

Next, we challenged the AG6 mice with these two ZL1 and ZL1(V2634M) viruses. Comparing with negative control group (PBS), both high and low dose (10^3^ and 10^2^ PFU per mouse) of inoculation could lead to significant weight loss and death to AG6 mice. However, the two groups inoculated with ZL1and ZL1(V2634M) show similar virulence in mice, (Fig. 7a–d). 7 days post virus inoculation, we collected various organs from mice infected with ZL1 and ZL1(V2634M) respectively. No significant difference in ZIKV RNA levels from various organs was observed between ZL1 and ZL1(V2634M) infected mice (Fig. 7e/f). These results suggest that both ZL1 and ZL1(V2634M) could cause death of infected AG6 mice efficiently, but the V2634M single amino acid substitution in NS5 did not change the virus pathogenesis in AG6 mice.

**Figure 8:**
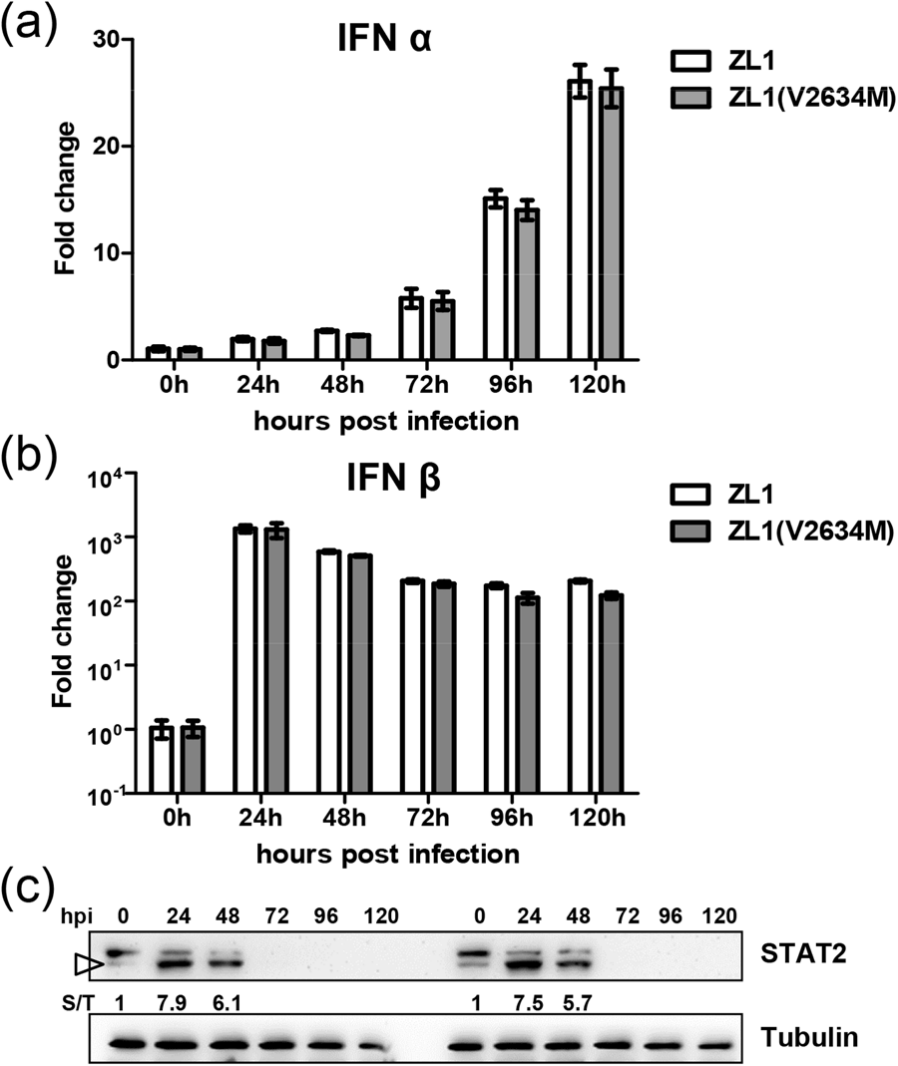
Comparison of IFN responses between wild type ZL1 and ZL1(V2634M). (**a**/**b**) Analysis of type I IFN responses in A549 cells. A549 cells were infected with ZL1 and ZL1(V2634M) virus(MOI = 0.01). Cell lysis were harvested at 0 h, 24 h, 48 h, 72 h, 96 h, 120 h post infection. IFNα/β RNA was detected by q-RT-PCR. Data represent the means of three independent assays; Error bars represent standard deviations from the means. (**c**) Western blot analysis of ZIKV NS5-induced STAT2 degradation in Vero E6. Vero E6 cells were infected with ZL1 and ZL1(V2634M) virus(MOI = 0.01). Cell pellets harvested at hours 0, 24, 48, 72, 96 h, 120 h post infection were detected by using anti-STAT2 antibody. Numbers indicate relative band intensity (STAT2/Tubulin, S/T) and targeted band for densitometry were indicated by triangle.

Since VeroE6 cells and AG6 mice were deficient in type I interferon (IFNα/β) production, we analyzed ZL1 and ZL1(V2634M) infection in A549 cells with low MOI(0.01) to compare induction efficiency of IFNα/β. Results showed that both ZL1 and ZL1(V2634M) could induce interferon mRNA abundance with similar efficiency (Fig. 8a and b). ZIKV antagonizes type I interferon through NS5 resulted STAT2 degradation^40^, we further assessed whether V2634M substitution could alter NS5 function in STAT2 degradation. Western blot analysis indicated that both ZL1and ZL1(V2634M) viruses induced STAT2 degradation at day 3 post infection, suggesting that wild type and mutant NS5 had comparable ability to antagonize innate immunity (Fig. 8c).

## Discussion

Zika virus (ZIKV) has emerged over the last decades to cause recent outbreaks of human infection in Pacific Islands and Latin America^3,44,45^. Most of these infections are asymptomatic. However, infection can occasionally result in Guillain-Barré(GBS) syndrome in adults and a wild range of severe congenital defects and malformations, including microcephaly^13–21,46^. Apart from acute infection, persistent infection of ZIKV was found in several human tissues (senital system, brain)^37,47–50^. Aiming to understand the molecular mechanisms which related to its replication, spread, pathogenesis and defining the viral molecular determinants of its evolution and transmission, we developed a straightforward reverse genetics system of an early Brizal epidemic ZIKV strain.

After several abortive attempts, we identified that the sequence 1–3000 nt was the major unstable region of ZIKA genome during the process of cloning. Previous studies used different strategies to overcome this bacterial toxicity of ZIKV genomes. Shan *et al*. generated a single-piece ZIKA infectious clone of Cambodia (FSS13025) by low-copy number vector^29^, Widman *et al*. and Weger-Lucarelli *et al*. constructed ZIKA infectious clone of strain PRVABC59, MR766, H/PF/2013, SPH2015 and BeH819015 by multiple piece system^32,33^. Liu *et al*. producted a ZIKA infectious clone of strain GZ01 by introns insertion^31^. In this study, we chose a different method that successfully worked for the construction of Dengue virus (DENV) and Japanese encephalitis virus (JEV) infectious clones^38^: silent mutation of ZIKA genome (1–3000 nt) to eliminate the activity of predicted bacterial promoters. Silent mutation of 8 predicted bacterial promoters with a total of 17 nucleotides substitutions conferred the infectious clone (pZL1) good stability in bacteria. After five times of serial plasmid transformation, sequencing results of recovered plasmid showed no point mutation (data no shown). It is unlikely that these substitutions change significantly the ZIKV genome structure for following reasons: (1) electroporation of ZL1 *in vitro* transcripts led to high virus (about 10^6^ PFU/ml at day 8 post electroporation of Vero E6 cells); (2) Similar to many clinical isolates^23,29,32^, ZL1 virus can infect multiple cell lines and replicate with comparable efficiency; (3) ZL1 infection of AG6 mice caused comparable pathogenesis characteristics. Altogether, our data confirmed that elimination of bacterial promoter activity in ZIKA genome is a simple and efficient way to construct infectious clone.

Liu *et al*. have proved a spontaneous mutation (A982V) in NS1 protein which could increase NS1 antigenaemia and promote ZIKA infectivity^23^, and Yuan *et al*. also found a functional adapted mutation (S139N) in prM protein make ZIKV more virulent to human NPCs and increased incidence of microcephaly^24^. These data confirmed that evolutionary mutation in ZIKA genome could contribute to recent ZIKV epidemics. Phylogenetic studies of African-Asian/Pacific and south America virus isolates proposed that M2634V mutation in NS5 emerged after ZIKA outbreak in south America in 2015 might be associated with increased emergence of ZIKV infection and accompanied neurological complications^25^. V2634 is located at the interface between methytransferase and RdRp domain, which might suggest a putative role of this mutation in genome translation and replication. However, V2634M reverse mutation did not alter replication efficiency of subgenome replicon replication. In order to test whether this fixed mutation may contribute the complete ZIKV life cycle in cell culture and ZIKV pathogenesis, we constructed a recombinant ZIKV based on the ZIKV infectious clone pZL1. Both ZL1 and ZL1(V2634M) could infect Vero E6, C6/36 and U-251 MG cell lines. However, there are no significant differences in viral replication and production *in vitro*. Interestingly, kinetics of ZIKV replication, viral protein expression and infectious progeny production in U-251 MG cells is approximately 2 days faster than that in Vero E6 and C6/36 cell lines, which may account for the preferred brain tropism and pathogenesis^42,43^. Additionally, the two viruses ZL1 and ZL1(V2634M) also showed comparable NS5 expression resulted proteasomal degradation of STAT2 and demonstrated similar tissue tropism virulence in AG6 mice and.

Over all, we present in this study a convenient, stable and robust infectious clone of an early Brazil ZIKV isolate. Reverse mutation V2634M did not cause significant impact on virus life cycle in multiple cell lines and on ZIKV tissue tropism and virulence in AG6 mice. We attempt to propose that ZIKV genome mutations acquired before 2013 confers the capability of ZIKV in causing ZIKV epidemics in Latin America and associated neurological complications.

## Electronic supplementary material

Supplementary Figures
