# Supplementary material for: Negligible contribution of M2634V substitution to ZIKV pathogenesis in AG6 mice revealed by a bacterial promoter activity reduced infectious clone
Source: Sci Rep. 2018 Jul 12;8:10491. doi: 10.1038/s41598-018-28890-0 (PMC6043478; doi:10.1038/s41598-018-28890-0)

Negligible contribution of M2634V substitution to ZIKV pathogenesis in AG6 mice revealed by a bacterial promoter activity reduced infectious clone

Fanfan Zhao,[1] Yongfen Xu,[1] Dimitri Lavillette,[1] Jin Zhong,[1]

Gang Zou[1] and Gang Long[1,*]

[1]Key Laboratory of Molecular Virology and Immunology, Institut Pasteur of Shanghai, Chinese Academy of Sciences, Shanghai, China.

*Corresponding author. Mailing address: Life Science Research Building, 320 Yueyang Road, Shanghai 200031, P.R. China. Phone: +86-21-54923161. Fax: +86-21-54923161. E-mail: glong@ips.ac.cn

Figure 2G

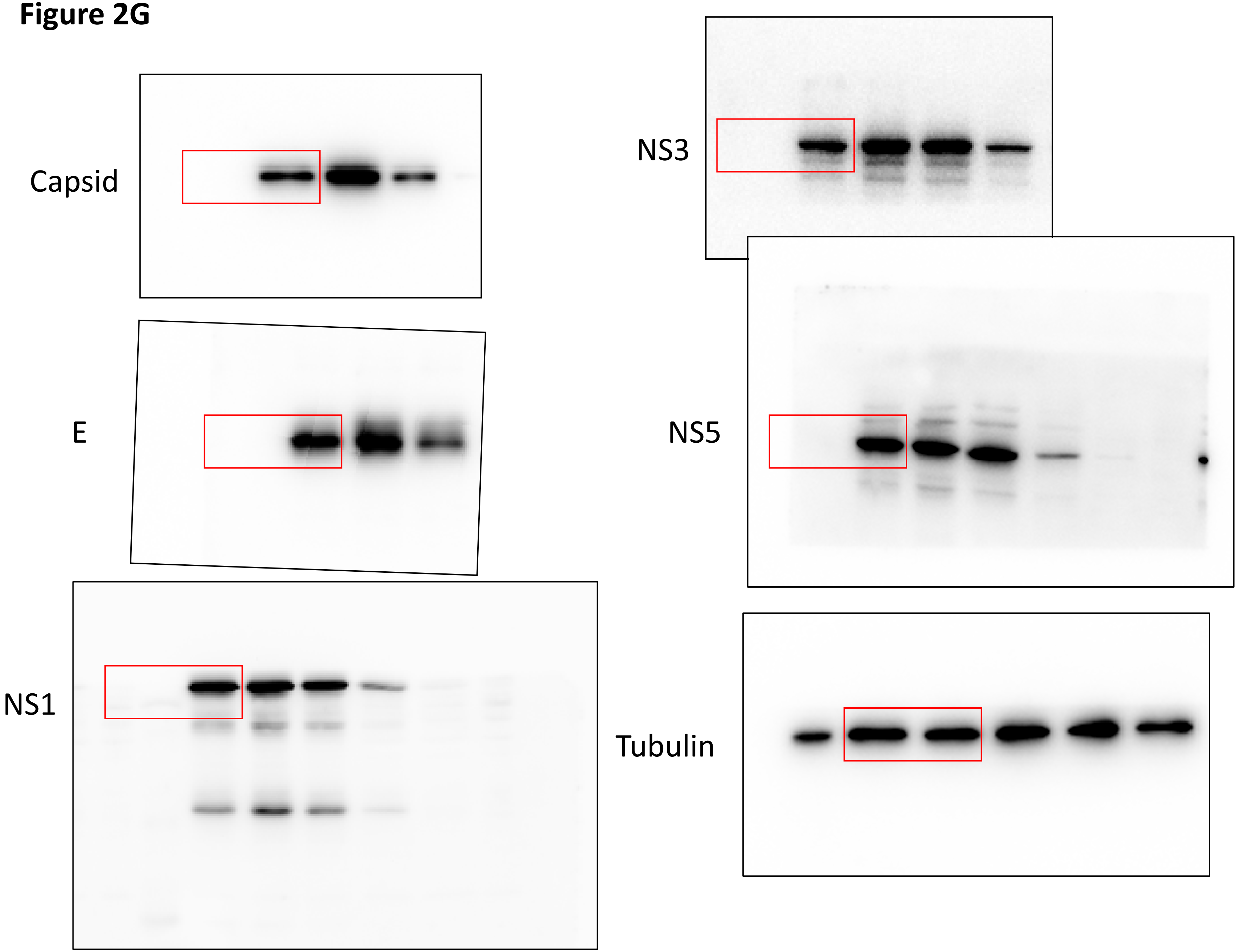

Figure 5D

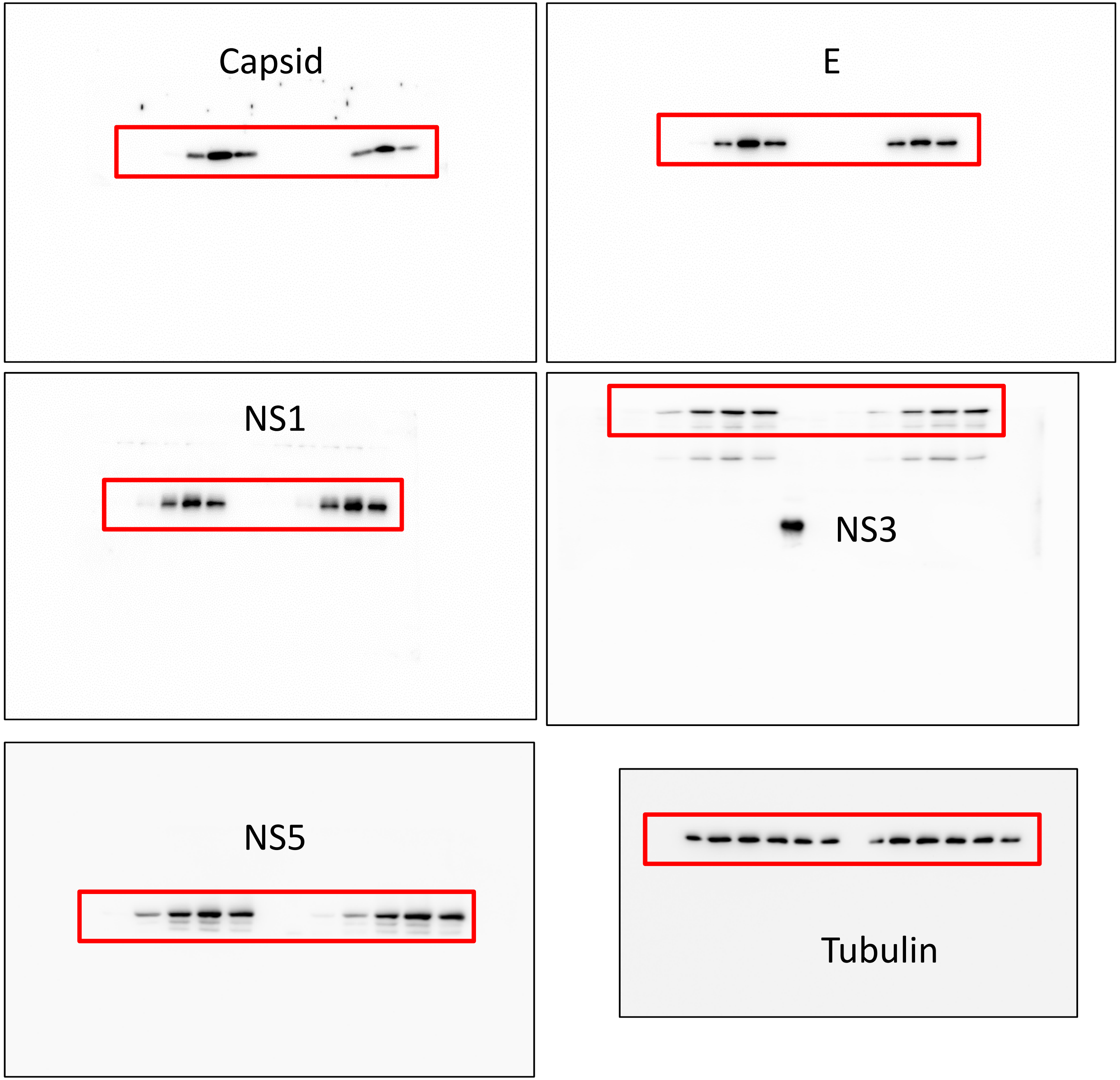

Figure 6C

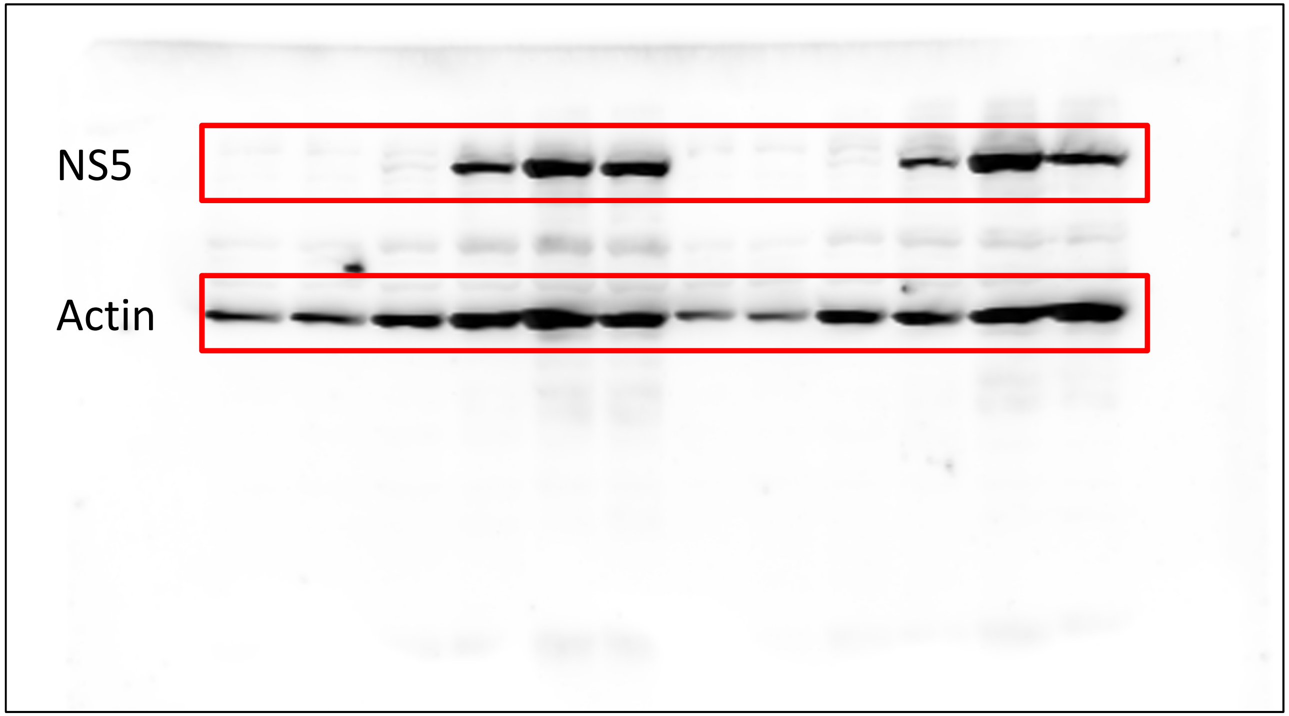

Figure 6F

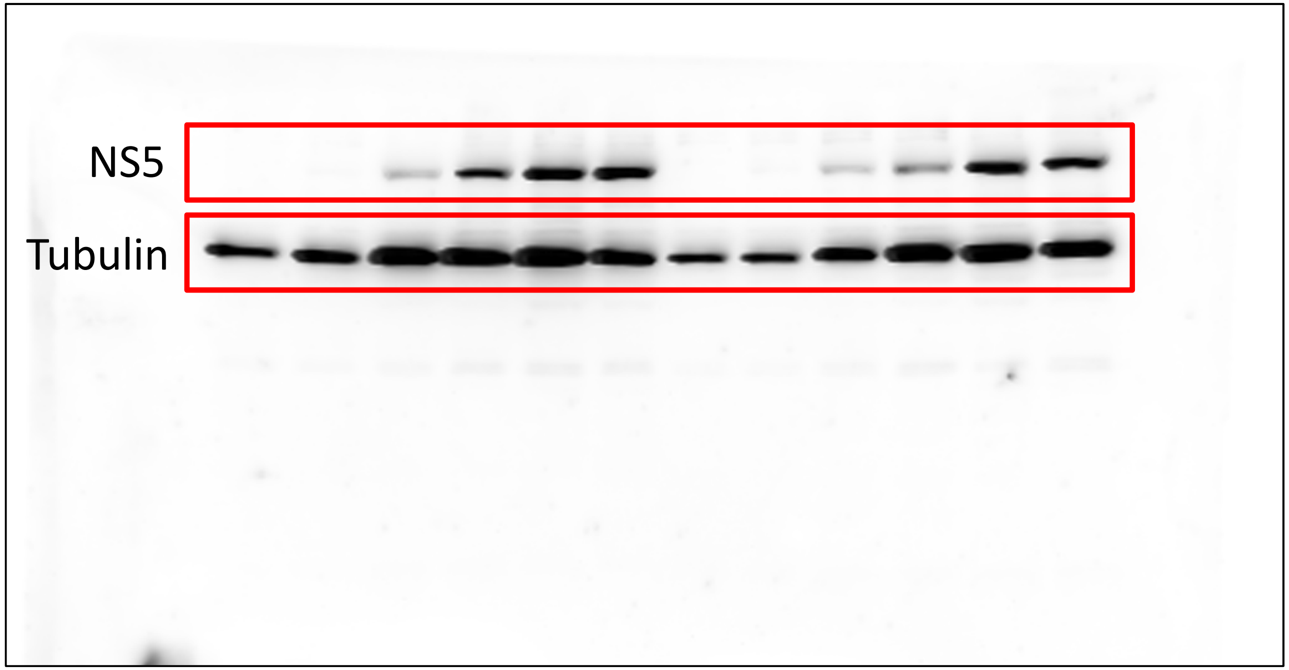

Figure 8 C

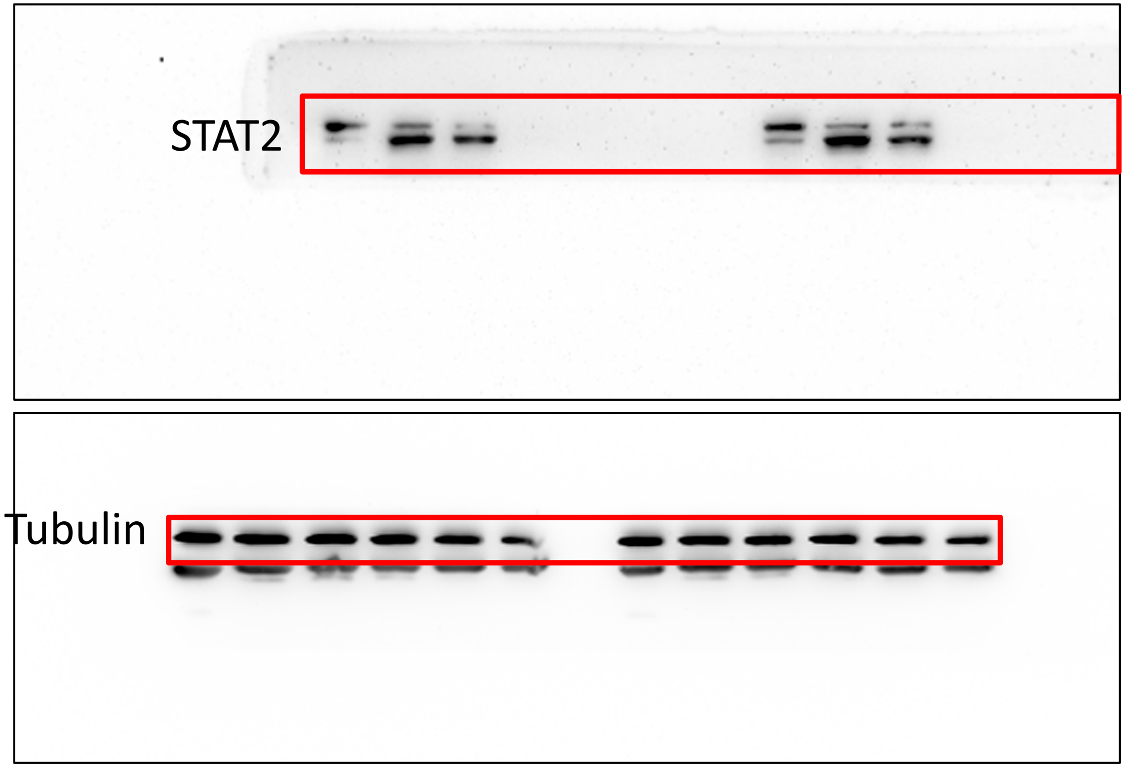

Supplement: Supplementary file 1 — Supplementary Figures [file 41598_2018_28890_MOESM1_ESM.pdf]
